# Micro versus Macro Shear Bond Strength Testing of Dentin-Composite Interface Using Chisel and Wireloop Loading Techniques

**DOI:** 10.3390/dj9120140

**Published:** 2021-11-30

**Authors:** Ahmed M. Ismail, Christoph Bourauel, Ahmed ElBanna, Tarek Salah Eldin

**Affiliations:** 1 Biomaterials Department, Faculty of Dentistry, Ain Shams University, Cairo 11566, Egypt; doctorbanna@asfd.asu.edu.eg (A.E.); prof_tareksalah@hotmail.com (T.S.E.); 2 Oral Technology Department, School of Dentistry, University of Bonn, 53111 Bonn, Germany; bourauel@uni-bonn.de

**Keywords:** shear bond strength, microshear bond strength, fractography, failure mode, chisel loading, wireloop loading, dental adhesive resin, dentine substrate, composite resin materials

## Abstract

Shear bond strength (SBS) testing is a commonly used method for evaluating different dental adhesive systems. Failure mode analysis provides valuable information for better interpretation of bond strength results. The aim of this study was to evaluate the influence of specimen dimension and loading technique on shear bond strength and failure mode results. Eighty macro and micro flowable composite cylindrical specimens of 1.8 and 0.8 mm diameter, respectively, and 1.5 mm length were bonded to dentinal substrate. Four study groups were created (*n* = 20): Macroshear wireloop, Gp1; Microshear wireloop, Gp2; Macroshear chisel, Gp3; and Microshear chisel, Gp4. They were tested for SBS using chisel and wireloop loading devices followed by failure mode analysis using digital microscopy and SEM. Two- and one-way ANOVA were used to compare stress at failure values of different groups while the Kruskal–Wallis test was used to compare between failure modes of the tested groups. Gp4 recorded the highest mean stress at failure 54.1 ± 14.1 MPa, and the highest percentage of adhesive failure in relation to the other groups. Specimen dimension and loading technique are important parameters influencing the results of shear bond strength. Micro-sized specimens and chisel loading are recommended for shear testing.

## 1. Introduction

Over the years, different direct restorative materials have been used in dentistry. Recently, there is an increasing demand for esthetic restorations, and hence, composite has gained a specific attention in restorative dentistry. Although esthetics is a crucial property for dental materials, specific concern should be given to the mechanical properties and bonding longevity of different restorations along with testing techniques and methodologies that determine the efficacy of bonded interfaces [1,2,3,4]. The functionality of dental materials should be assessed by different testing techniques. One of these methods is to evaluate the ability of a material to bond to a substrate through different bond strength tests [5].

Despite the noticeable advances achieved in adhesive dentistry in the past 5 decades, the bonded interface is still considered as the weakest point of an adhesive restoration [6,7]. Laboratory tests can gather data quickly and easily about a specific parameter or property. It is possible to measure one specific parameter while maintaining all other variables constant. Different experimental groups can be tested simultaneously within the same study set-up [8]. Although the relationship between bond strength test results and reliability of clinical performance for dental adhesives remains questionable, recent evidence proves that clinical reliability can, to some extent, be estimated based on laboratory results [9,10,11].

Clinical trials are the most accurate tests to evaluate adhesive dental restorations. Unfortunately, however, they cannot identify the exact reason of failure due to the simultaneous integration of different complex stresses on restorations within the challenging oral cavity environment [8]. Moreover, because of the continuous and rapid evolution of new materials, there is more dependance on the properties of a dental material that can be retrieved from the laboratory research, as the process of gathering clinical evidence takes such a long time that the material may be totally replaced on the dental market by the time clinical results evolved. Thus, the collection of laboratory data is often justified as being driven by the intension to use such data to predict the in vivo clinical performance [12,13,14].

Owing to the simplicity of testing procedures, SBS testing is considered as one of the most commonly used methods for bond strength measurement, and the results of the measured SBS with various conditions are reported in the literature [15]. Advantages of shear tests include ease of specimen preparation, simple testing protocol, and lower incidence of pretest failure. However, concerns regarding consistency of the obtained measurements arose, as cohesive failures within the substrate or composite were observed with newly introduced adhesives. The explanation for this fact was that stresses were mostly concentrated in the tooth substrate or composite, hence causing its premature failure before failure of the adhesive interface itself [16].

Studies using finite element analysis demonstrated that conventional (Macro) shear bond strength test results in non-uniform and heterogenous stress patterns [17]. The need for novel methods overcoming these limitations led to the evolution of specimens with small bonding areas (i.e., less than 1 mm^2^), in what is known as microshear bond strength tests [11]. Earlier shear test methods used large bonding areas; however, higher bond strength and incidence of adhesive failures were observed with smaller bonded areas [18]. Knife edged chisel was the traditional loading method proposed by ISO standards, despite having a lot of concerns regarding stress concentration at a specific point on the bonded interface, leading to complex representation of stresses and underestimated bond strength values. Wireloop methods have been also utilized to de-bond the specimen in SBS tests [19].

Although there are numerous studies investigating the bond strength of various interfaces, so far, there is no standardized recommended protocol for bond strength assessment [20]. Failure mode analysis can provide highly valuable information for the detection of weaknesses in different testing methodologies so as to improve their reliability, providing results that represent the actual strength of adhesive junction. The aim of this study was to evaluate the influence of specimen dimension and loading technique on dentine-composite shear bond strength. The null hypothesis was that there is no effect of neither specimen dimension nor the loading technique on SBS results.

## 2. Materials and Methods

### 2.1. Specimen Preparation

Ten sound human lower wisdom teeth (disinfected with thymol 0.1% for 30 days after extraction) from the Dental Biomaterials Teeth Storage Bank, Ain Shams University, Cairo, Egypt (Ethical Committee approval no. FDASU-RECIM 011923), were transversally sectioned using a diamond disc and a straight hand piece at a low speed under air–water coolant into two pieces at the intermediate dentin level midway between the central occlusal fossa and cementoenamel junction. Radicular tooth portion was sectioned transversally at the cementoenamel junction, yielding twenty tooth halves. Polypropylene (PP) tubes with 1.8 cm internal diameter and 1 cm height were used as molds in which the sectioned teeth were placed and embedded in cold cured acrylic resin. Each sectioned tooth fragment was placed inside a PP mold with its flat surface touching a glass slab. Acrylic resin was packed into the PP mold surrounding the sectioned tooth.

After setting, the flat surface of the tooth was finished and polished with ascending grits (150 and 320 µm) of silicon oxide sandpaper for artificial smear layer simulation [21]. Silicon tubes of 1.8 and 0.8 mm internal diameters were sectioned transversally in 1.5 mm intervals to act as molds for the flowable composite used for shear and microshear bond strength testing, respectively. Universal adhesive (All Bond Universal, BISCO, Inc, Chicago, IL, USA, LOT. 1900006405) was applied actively in self-etching mode to the polished dentinal surface, followed by a gentle air jet for 10 s according to the manufacturer’s instructions. Four tubes—either 1.8 or 0.8 mm—were placed over the bond-coated dentinal surface before light curing. Bond curing was achieved for 20 s using a light curing unit (3M ESPE Elipar S10, USA).

**Figure 1:**
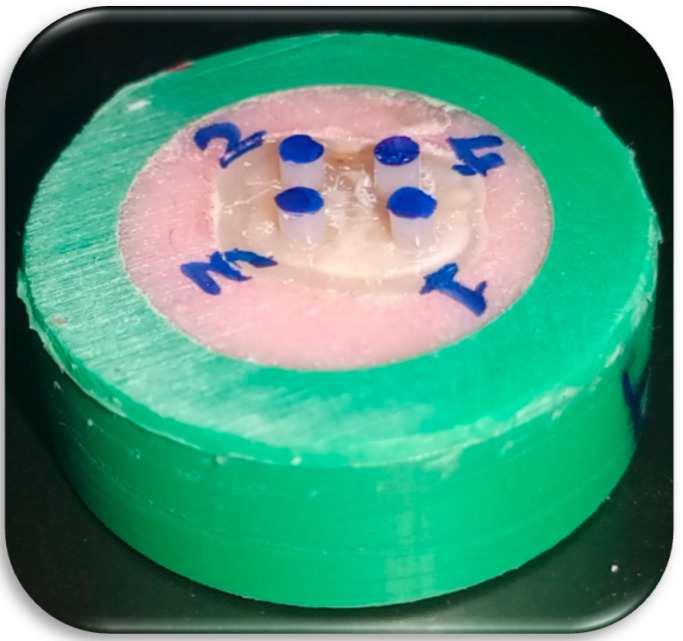
Four macroshear cylindrical composite samples bonded to dentinal substrate.

Five tooth halves (dentinal substrates) were used for each group (n = 20). Flowable composite (Polofil NHT flow, VOCO, Cuxhaven, Germany, LOT. 1940539/1941379, Shade A2) was injected into the silicone tubes from the bottom upwards to avoid bubbles, then covered with transparent mylar strip and cured for 20 s according to the manufacturer’s instructions. Silicone molds were then cautiously vertically sectioned, using a sharp lancet to avoid pretest failures, yielding four composite stubs bonded to the dentinal substrate (Figure 1).

Specimens were stored in distilled water at 37 °C in an incubator for 24 h until testing. Half of the specimens were tested using chisel and the other half were tested using wireloop loading devices.

### 2.2. Specimen Grouping

A total of 80 specimens were randomly allocated into 4 groups (*n* = 20) according to specimen diameter and loading device, as follows:

Macroshear wireloop ➔ Grp (1)
Microshear wireloop ➔ Grp (2)
Macroshear chisel ➔ Grp (3)
Microshear chisel ➔ Grp (4)

### 2.3. Specimen Loading

**Figure 2:**
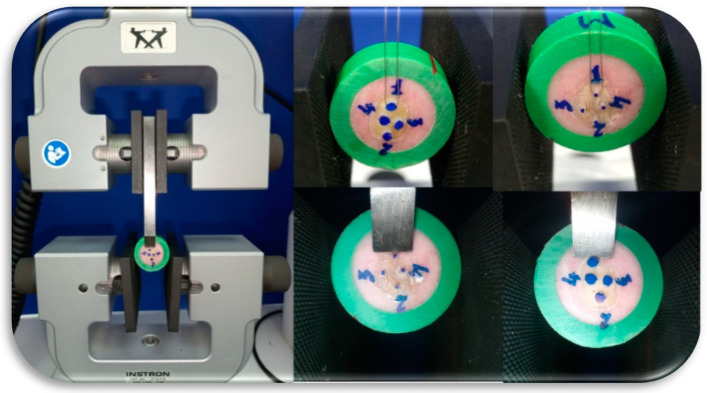
Macro- and microshear composite samples loading using chisel and wireloop.

All specimens were tested using an Instron 3365 (Norwood, MA, USA) universal testing machine, at a crosshead speed of 1 mm/min (Figure 2). Stress at failure values were recorded for both shear and microshear samples using chisel and wireloop as loading devices. After specimen debonding, each cylindrical composite stub diameter was measured using a digital caliper to detect any diameter variabilities from the silicon mold. Stress at failure values were calculated according to the measured specimen diameter.

### 2.4. SEM Fractographic Analysis

All corresponding samples (dentinal substrates and fractured composite stubs) were collected after bond strength testing for fractographic analysis. They were first examined under a digital microscope (50×, Dino-lite, New Taipei City, Taiwan) and then prepared for scanning electron microscopy (SEM) examination. Samples were alcohol degreased for 2–3 min using an ultrasonic vibrator (Bandelin, Berlin, Germany), then fixed to SEM aluminum pin stubs using double-face conductive carbon tabs (PLANO Leittabs, Ernst, Germany) and conductive carbon cement (PLANO Leit C, Ernst, Germany). Specimens were cement-coated from the bottom upwards to be rendered conductive, then placed in silica gel particles to absorb moisture. Samples were then platinum sputter-coated (HHV scancoat six, West Sussex, UK). Argon gas was added as an adjunctive aid with the low-vacuum sputter machine for better sputtering process. All samples were finally examined using high-vacuum SEM (EDAX camscan S4, Mahwah, NJ, USA) under different magnifications (from 35× to 4000×).

### 2.5. Statistical Analysis

Sample size was estimated based on data collected from a pilot study for the 4 groups (*n* = 10). Large effect size (f = 0.89) was determined based on the calculated means and standard deviation of 16.15 within groups. A minimum sample size of 24 (*n* = 6 for each group) was estimated to be enough to achieve 96% power to detect with a 0.05 significance level [22]. Sample size was increased to 80 (*n* = 20) for statistical analysis reliability.

Data were explored for normality using the Kolmogorov–Smirnov test. Shear bond strength data showed a parametric distribution. Two-way ANOVA was used to show the effect of specimen size and applicator design followed by multiple comparison with Bonferroni’s adjustment. One-way ANOVA was used to compare between all groups followed by Tukey’s HSD test for pairwise comparisons. The significance level was set at *p* < 0.05. For fractographic analysis, the Kruskal–Wallis test was used to compare between tested groups followed by multiple comparisons with the Dunn–Bonferroni adjustment. Statistical analysis was performed with IBM^®^ SPSS^®^ (SPSS Inc., IBM Corporation, Armonk, NY, USA) Statistics Version 26 for Windows.

## 3. Results

### 3.1. Shear Bond Strength

**Figure 3:**
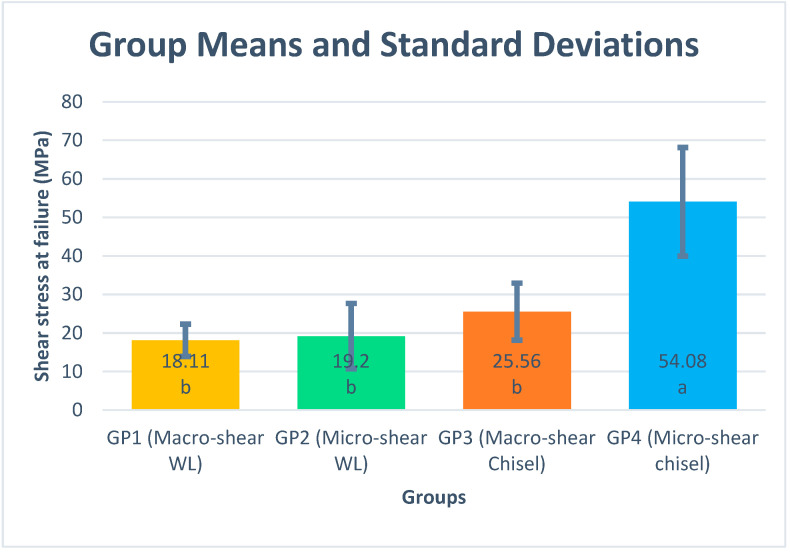
Means and standard deviations for different tested groups. Different letters within the mean column indicate significant differences based on Tukey’s HSD post hoc test (adjusted *p* < 0.05).

Shear bond strength results are presented as mean and standard deviation for each group (Figure 3). Specimen dimension showed a significant effect on bond strength results using the chisel loading technique (*p* value < 0.001), while upon using the wireloop, no significant effect was noticed (*p* = 0.711). Different loading techniques showed a significant effect on bond strength regardless of the specimen dimension; *p* values for macro- and micro-sized samples were 0.013 and < 0.001, respectively. One-way ANOVA showed that the Group 4 results were significantly higher than that of the other three test groups (Figure 3). Different applicator designs and specimen dimensions showed a significant effect on the shear bond strength (*p* < 0.001). Similarly, the interaction between both variables showed a significant effect on shear bond strength (*p* < 0.001) (Table 1).

### 3.2. Failure Mode Analysis Results

**Figure 4:**
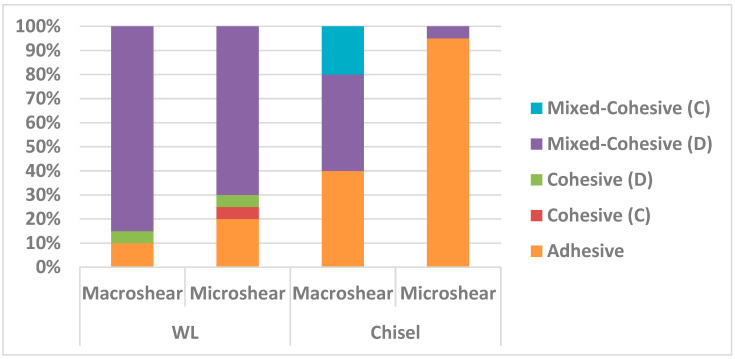
Stacked bar chart showing the failure mode percentage strength for different tested groups.

Different percentages of failure modes among the four groups are shown in Table 2. Group 4 recorded the highest percentage of adhesive failures and the least percentage of mixed failures amongst all the other groups. Regarding the loading devices, the chisel showed more adhesive failure percentages compared to the wireloop. Moreover, wireloop loading method resulted in more pure cohesive failure percentages in comparison to the chisel one. Regarding specimen dimensions, the micro-sized specimens showed higher adhesive failure percentages compared to the macro-sized ones. By tracing the bar chart of adhesive failure percentages (Figure 4), it can be noticed that it showed nearly the same pattern as the mean stress at failure values among the four testing groups.

## 4. Discussion

The SBS test has long been criticized for not being appropriate to represent the so-called “actual” or “true” bond strength by shearing at the bonded interface. The crack tip leading to eventual fracture of the SBS test could be initiated by an undesired high-tensile component of stresses, rather than the minimum required shear stress. Unfortunately, the load is wasted in creating a cohesive failure within the substrate, rather than shear loading on the adhesive interface itself [5,19,23]. Although shear bond strength is one of the most commonly used bond strength tests, numerous studies, especially those with numerical stress analysis, state that the test lacks proper standardization and that the results obtained by the test are highly variable. Moreover, the stress pattern of the test is not uniform and unfortunately, in many cases, it does not represent the true shearing process [19]. Despite the limitations of the shear test, the ease of sample preparation, minimal laboratory equipment needed, lower incidence of pretest failure, ease of specimen alignment with the loading device, and overall non-technique sensitivity make it an extensively used method for the evaluation of dental adhesives [24,25]. There are several factors contributing to the variability in results retrieved from shear testing and the variation in a single factor may lead to a dramatic change in the final results. The existence of different loading techniques, specimen dimensions, cross head speeds, bonding protocols, substrates, and storage conditions make it extremely difficult to compare results retrieved from different SBS studies or combine them in single meta-analyses [26].

It was the aim of this study to measure the influence of specimen dimension and loading technique on shear bond strength testing methods. During specimen preparation, the self-etch bonding protocol was adopted rather than the etch and rinse technique to eliminate the subjectivity and technique sensitivity of the latter [27,28]. The same flowable composite was used for both shear and microshear samples to standardize all test parameters except for specimen dimension and loading technique. Four samples were bonded to the same dentinal substrate to standardize substrate variability as much as possible. Bonding was standardized at an intermediate dentin level to avoid differences in bond strength values between different dentinal layers [29]. Silicone molds were removed prior to testing since they would adversely affect testing results owing to their rubbery, flexible nature.

Knife-edged chisel was the traditional loading method proposed by ISO/TR 11405:1994 despite concerns regarding stress concentration at a specific point on the bonded interface, leading to complex representation of stresses and underestimated bond strength value. Wireloop methods have been also utilized to de-bond specimens in SBS tests. Orthodontic ligature wire of the smallest possible diameter (0.2 mm) was used to provide better adhesive interface engagement so as to be separated from the adhesive interface by a distance of 0.1 mm; it only touches the specimen at the surface tangent to the wire in relation to the specimen. Unfortunately, the wireloop can only be placed at a distance equal to its radius from the adhesive interface, and the point of contact is a curved surface produced by the wire; thus, it is impossible to achieve loading exactly at the interface when using the wireloop [19].

Composite stubs’ diameters were measured with a digital caliper after debonding to detect any dimension variabilities caused by flexible silicone tube molds, as it was difficult measure them while bonded to dentin without pretest failures. Stress at failure values were recalculated according to the measured diameter [16,30]. During testing, it was always taken into consideration that the loading device is perfectly aligned along with the dentinal flat surface parallel to the loading axis and perpendicular to the specimen.

One of the limitations noticed during testing was the repeated wire ductile failure with macroshear samples. We also noticed that macroshear samples took more testing time owing to wire ductile deformation or stretching prior to actual specimen loading. This limitation can subsequently be minimized by using a wire of larger diameter, but inevitably, it will have a greater radius of separation from the adhesive interface. Moreover, it was practically difficult to assure proper wire–adhesive interface engagement owing to the wire’s flexibility, so in most cases, it was expected to be separated from the adhesive interface by a distance even larger than the wire radius itself. Different measures were performed to enhance the consistency of SBS testing method. The length of the cylindrical sample (adherent) should be decreased as much as possible to avoid applying the load at a distance from the adhesive interface, and hence, bending rather than shearing will occur. A short sample length (from 1 to 1.5 mm) makes it easier for the operator to notice improper adhesive interface engagement by the wire [5].

The pattern of mean stress at failure values for the four groups was as follows, in descending order: Group 4, Group 3, Group 2, and Group 1. It was noticed that the Group 4 samples showed the highest stress at failure values (Figure 3). The mean stress at failure values were nearly the same for both shear and microshear wireloop-loaded samples and their values were significantly lower than the chisel-loaded ones. Lower stress at failure results for WL-loaded samples may be attributed to application of load at a distance from the adhesive interface equivalent to the wire radius or more, so that specimen loading occurs by bending rather than shearing [7,19], which lowers the force required to de-bond the adhesive interface and hence lowers the stress at failure results.

Sultan et al. [31] noticed that non-uniform stresses are usually associated with shear testing methods with predomination of tensile stresses rather than shear ones. This adversely affects the consistency of the testing technique since part of the loading force is consumed on disrupting the cohesive force of adhered material rather than shearing the adhesive interface itself, so the resultant value may not truly represent the strength of the adhesive interface but also the cohesive strength of substrate material [5,19,23]. Practically, the wireloop showed risk of deformation and slippage during specimen loading, especially the macro-sized ones. If more than a single wire is required to be used for testing because of wire failure, wire preadaptation on a cylindrical body having the same diameter as that of the specimen is mandatory to assure the same circumferential engagement with all specimens and avoid bias in results. Microshear samples showed higher difficulty in their preparation, taking longer time, needing higher manual dexterity for accurate composite injection, and having higher incidence of pretest failures during silicone mold removal or inadvertent handling. It is also known that the adhesive layer is mostly thicker in micro- rather than macro-sized samples [32]. However, the micro samples are thought to follow the all-or-none principle, so that if an air bubble is incorporated, pretest failure would be expected, which is preferred to avoid false positive results.

In this work, samples were first examined using a digital microscope for the purpose of accurate indexing of the complementary fractured surfaces. Achieving vacuum for sputtering and for SEM examination with tooth samples was more difficult than composite stubs since teeth samples are known to be moist from the inside, so samples were placed in a closed box with color-changing silica gel particles to absorb moisture.

Failure modes were classified as the following:

(a) Adhesive (between dentin and adhesive, between composite and adhesive, cohesive failure in the adhesive, mixed failure of previously mentioned modes);
(b) Mixed-Cohesive (D) (adhesive and cohesive in dentin);
(c) Mixed-Cohesive (C) (adhesive and cohesive in composite); and
(d) Pure cohesive (in dentin or composite).

**Figure 5:**
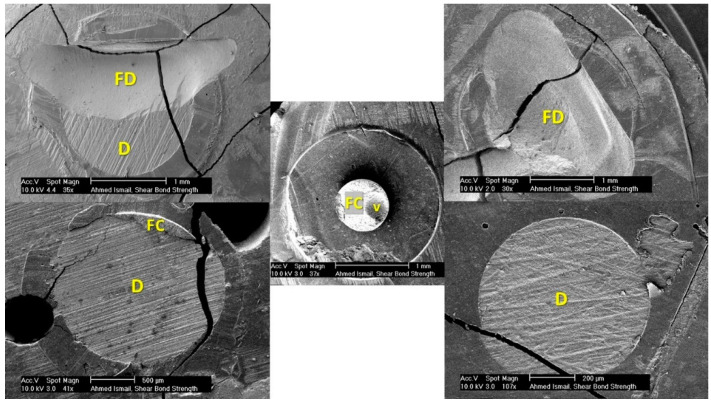
SEM images showing different failure modes in dentinal substrates. Top right: Cohesive (D); top left: Mixed-Cohesive (D); bottom right: Adhesive; bottom left: Mixed-Cohesive (C); center: Cohesive (C). D = dentin; V = void; FD = fractured dentin; FC = fractured composite.

**Figure 6:**
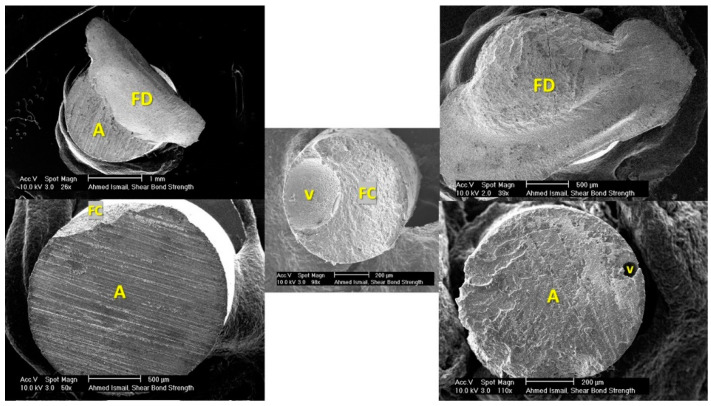
SEM images showing different failure modes in the corresponding composite; A = adhesive; V = void; FD = fractured dentin; FC = fractured composite.

Both dentinal substrate and composite stub were examined under a microscopes since examining the two complementary fractured surfaces provides better affirmation of the failure mode (Figure 5 and Figure 6). Some cracks were noticed in the sputter coat but without affecting the surface topography and failure assessment. Fractography and failure mode analysis showed high susceptibility to cohesive failures in bonded areas located far from the load application points, i.e., in the upper half of wireloop-loaded samples and in the lower half of chisel-loaded samples. It seems that wherever the loading device is touching the outer perimeter of the specimen, controlled adhesive debonding occurs, while at circumferential points where no touching occurs, complex stresses are generated, leading to cohesive failure in dentin, taking the shape of a mussel (Figure 5 and Figure 6, top left).

The same shape was noticed in mixed failures presented by Andrade et al. [33] and Chai et al. [14], but it was not described as being mussel-shaped. The mussel shape was noticed to have its vertex pointing at the starting point of cohesive failure. This pattern was noticed in cohesive failures associated with both macro- and microshear samples loaded by chisel and wireloop, but it was more frequent and obvious in the macroshear and WL-loaded ones. Sometimes a ditch defect was noticed in composite stubs loaded by chisel, indicating the chisel pushing point over the specimen. More mixed adhesive and cohesive failures in dentin were noticed especially in the wireloop-loaded groups, which may be attributed to the inevitable bending caused by wireloop loading. There are several factors jeopardizing the reliability of wireloop loading method: the wire deformation, which was highly observable with macroshear specimens; the unavoidable bending caused by loading at a distance that is at least equal to or more than the radius of the debonding wire; the possibility of wire slippage during testing; the difficulty to assure proper specimen alignment with the loading device owing to the wire flexibility, simply carried out in chisel loading by aligning the substrate flat surface with the chisel’s flat edge; and finally, the possibility of unequal circumferential engagement if more than one wire is used, since exact circumferential adaptation can never be assured. Microshear samples showed higher adhesive failure percentages in comparison to the macro-sized ones, and this may be attributed to the smaller specimen diameter, which reduces the chance of complex stress generation, yielding more adhesive rather than mixed failures.

In our experiment, micro samples showed higher adhesive failure percentages than the macro ones, despite having higher mean stress at failure values, which was slightly confusing. It was expected that the higher the mixed or cohesive failure percentages, the higher would be the mean stress at failure results, as stresses have to disrupt the cohesive bonds of substrate material itself [5,7,19,23]. This may be attributed to the fact that failure by bending occurs at lower force levels than failure by shearing, and hence, stress at failure values for samples subjected to bending, i.e., wireloop-loaded samples, will logically be lower than those subjected to failure by shearing, which is in agreement with a study published by van Meerbeek et al. [7]. In this study, we found that considerable bending rather than shearing was noticed with wireloop rather than chisel loading.

By observing the bar chart of adhesive failure percentages, it can be noticed that the four test groups showed nearly the same sequential pattern as the mean stress at failure values. This may affirm that mixed and cohesive failures are mostly resulting from the bending action of the loading device over the specimen, which can de-bond the specimens at lower stress levels than those needed to shear the specimen off the substrate, even if the cohesive force of substrate material is sharing in failure resistance.

## 5. Conclusions

Within the limitations of this study, we can conclude that:

- Loading technique and specimen dimensions are sensitive factors influencing shear bond strength and failure mode analysis results.
- Microshear bond strength test is more recommended than the macroshear one, whenever feasible.
- Chisel loading is more recommended than wireloop.

## Figures and Tables

**Table 1:** Two-way ANOVA showing the effect of size and applicator design on mean shear bond strength.

Source	Type III Sum of Squares	df	Mean Square	F	Sig.
Size	4385.982	1	4385.982	51.208	<0.001 *
Design	8962.320	1	8962.320	104.639	<0.001 *
Size X Design	3764.082	1	3764.082	43.947	<0.001 *
Error	6509.410	76	85.650		
Total	91,993.688	80			
Corrected Total	23,621.793	79			

* = Significant.

**Table 2:** Frequency and percentage for different tested groups.

	Adhesive		Cohesive (C)		Cohesive (D)		Mixed-Cohesive (D)		Mixed-Cohesive (C)		Rank	*p*-Value
	N	%	N	%	N	%	N	%	N	%		
Macroshear (WL)	2	10.0%	0	0.0%	1	5.0%	17	85.0%	0	0.0%	a	<0.001 *
Microshear (WL)	4	20.0%	1	5.0%	1	5.0%	14	70.0%	0	0.0%	a	
Macroshear (Chisel)	8	40.0%	0	0.0%	0	0.0%	8	40.0%	4	20.0%	a	
Microshear (Chisel)	19	95.0%	0	0.0%	0	0.0%	1	5.0%	0	0.0%	b	

* = Significant. Different letters within the rank column indicate significant differences based on the Dunn–Bonferroni adjustment (adjusted *p* < 0.05).
